# Posttranslational Regulation of the NLR Family Pyrin Domain-Containing 3 Inflammasome

**DOI:** 10.3389/fimmu.2018.01054

**Published:** 2018-05-18

**Authors:** Do-Wan Shim, Kwang-Ho Lee

**Affiliations:** ^1^Department of Applied Life Science, Graduate School, Konkuk University, Chungju, South Korea; ^2^Department of Biotechnology, College of Biomedical and Health Science, Research Institute of Inflammatory Disease, Konkuk University, Chungju, South Korea

**Keywords:** NLR family pyrin domain-containing 3, inflammasome, posttranslational regulation, ubiquitination, phosphorylation, alkylation, S-nitrosylation

## Abstract

The NOD-like receptor family pyrin domain-containing 3 (NLRP3) inflammasome is a multi-protein complex that can be activated by a variety of pathogen-associated molecular patterns or damage-associated molecular patterns. Inappropriate NLRP3 inflammasome activation can induce autoinflammatory, autoimmune, or metabolic disorders. Therefore, NLRP3 is an attractive target against NLRP3 inflammasome activation, and specific targeting of NLRP3 might be an intriguing approach to the development of drugs for the treatment of NLRP3 inflammasome-related diseases. Although many studies with varied mechanistic approaches were reported in inhibition of NLRP3 inflammasome activation, mechanisms related to regulation of posttranslational modification (PTM) of NLRP3, as a focal point has not been thoroughly addressed. Recently, extensive investigations of PTMs of NLRP3 have led to partial understanding of the mechanisms involved in NLRP3 inflammasome activation. In this review, we focused on the role of PTMs regulating NLRP3 inflammasome activation.

## Introduction

Inflammasomes are unique, complex structures formed by proteins in cytosol. Inflammasome activation results in the recruitment and activation of caspase-1, which then cleaves pro-IL-1β and pro-IL-18 into their active forms. Inflammasomes are characterized by a particular sensor or receptor molecule, such as absent in melanoma 2 (AIM2), IFNγ-inducible protein 16 (IFI16), and various Nod-like receptor (NLR) subsets (1, 2). Among a number of inflammasomes, the NLR family pyrin domain (PYD)-containing 3 (NLRP3) inflammasome is the most well studied, because it responds to infectious pathogens, such as Sendai virus, adenovirus, influenza virus (3), *Escherichia coli, Staphylococcus aureus* (4), and host-derived damage-associated molecular patterns, including extracellular adenosine triphosphate (ATP) (5), monosodium urate (MSU) crystals (6), cholesterol (7), silica (8), aluminum salts (9), amyloid deposits (10), and fatty acids (11).

The autoinflammatory syndromes associated with gain-of-function mutations of NLRP3 leads to abnormal NLRP3 inflammasome activation causing cryopyrin-associated periodic syndromes (CAPS), a group of rare, inherited, auto-inflammatory diseases, such as Muckle–Wells syndrome, familial cold urticaria, and neonatal onset multisystem inflammatory disease (NO-MID) (12, 13). Reports also revealed that excessive activation of NLRP3 inflammasome could play an important role in other diseases, such as multiple sclerosis (14), Alzheimer’s disease (15), metabolic disorders, such as gout (6), atherosclerosis (16) and type 2 diabetes (11, 17–19). NLRP3 comprises a PYD, a nucleotide-binding domain (NBD), and leucine-rich repeat (LRR) motif (20). The NLRP3 inflammasome is a multi-protein complex mainly composed of NLRP3, ASC, and caspase-1 (21). Therefore, NLRP3 is an ideal target for the development of specific inhibitors against the NLRP3 inflammasome.

There have been many efforts to develop inhibitors specific to the NLRP3 inflammasome by targeting NLRP3. Strategies directing toward posttranslationally modified NLRP3 are particularly intriguing methods in developing NLRP3-specific inhibitors.

Posttranslational modifications (PTMs), which are carried out by covalent bonding of low-molecular weight groups, such as alkyl groups, phosphate groups, or the ubiquitin protein, to amino acids, are closely related to numerous physiological activities of proteins. PTMs were also known to control immune responses through the regulation of protein folding, location, stability, and interaction with other molecules (22, 23). Many types of PTMs, such as phosphorylation, ubiquitination, alkylation, and S-nitrosylation have been detected in NLRP3, and these PTMs play important roles in NLRP3 inflammasome activation (24). In this review, we focus on how individual PTMs are involved in NLRP3 and regulate NLRP3 inflammasome activation and its subsequent functional roles.

## NLRP3: Phosphorylation

Phosphorylation of NLRP3 was first reported by Spalinger et al. (25), and so far, five different phosphorylation sites have been proposed (25–30).

### Protein Tyrosine Phosphatase Non-Receptor 22 (PTPN22)

**Figure 1:**
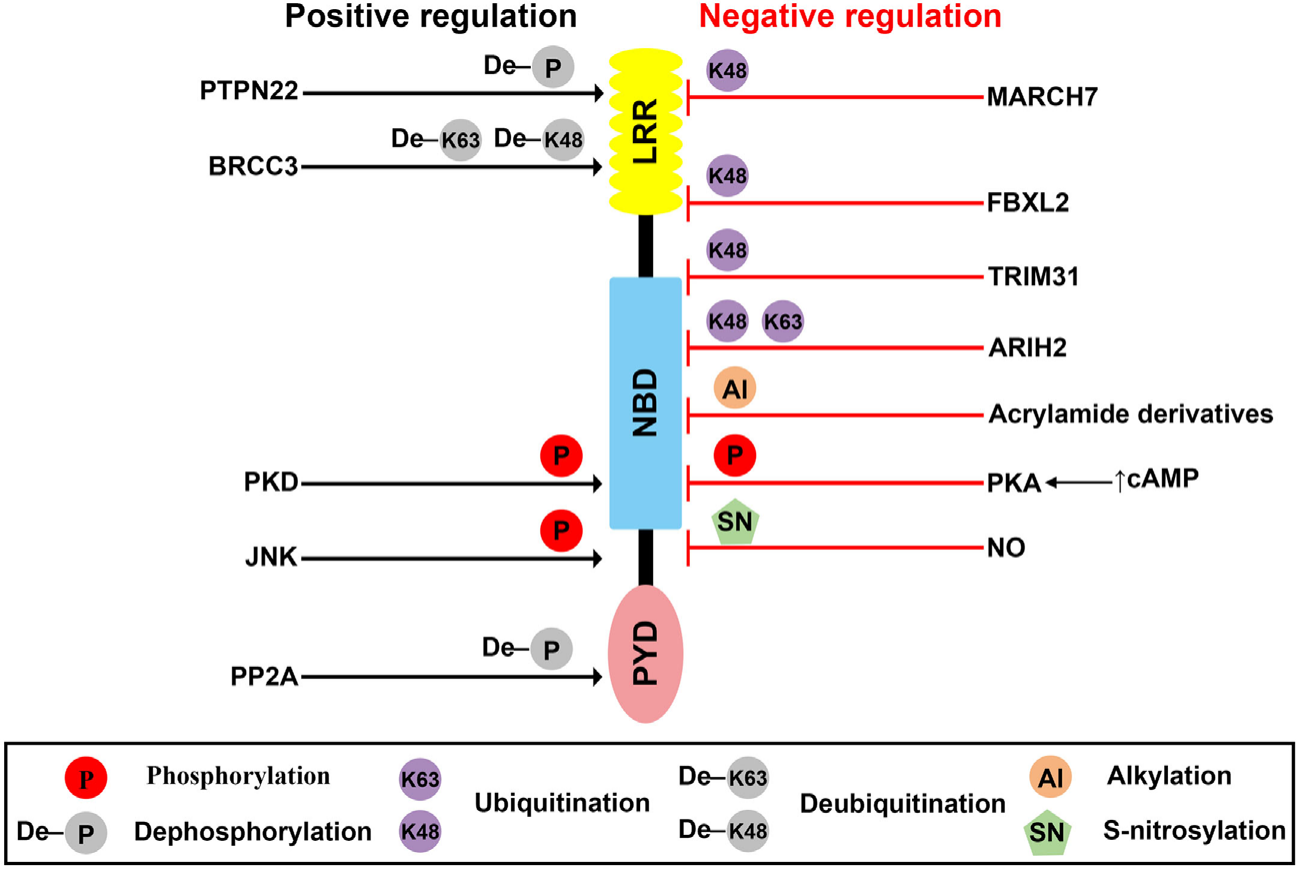
Posttranslational modification (PTMs) of NLR family pyrin domain (PYD)-containing 3 (NLRP3). Schematic of NLRP3 structure and mechanisms of posttranslational regulation of the NLRP3 inflammasome are represented. PTMs that promote activation of the inflammasome are shown as black arrows, whereas those that suppress inflammasome activation are shown as red blocks.

The PTPN22, a protein tyrosine phosphatase, is known to be associated with several inflammatory disorders, including Crohn’s disease (31), rheumatoid arthritis (32), systemic lupus erythematosus (33), and type-1 diabetes (34). Recently, Spalinger et al. reported that PTPN22 positively regulates NLRP3 inflammasome activation through its phosphatase activity, but does not similarly regulate AIM2 and NLRC4 inflammasome activation (25). Phosphorylation of the Y861 residue at the LRR domain of NLRP3 negatively regulates NLRP3 inflammasome activation, but NLRP3 inflammasome stimulators, such as ATP, MSU crystals, and silica crystals, induce tyrosine dephosphorylation of NLRP3 at Y861 in a PTPN22-dependent manner, leading to activation of NLRP3 (25). PTPN22 directly interacts with NLRP3 in NLRP3 inflammasome-activated macrophages (25). PTPN22 deficiency prevents MSU-induced peritonitis in mice by inhibition of inflammasome-dependent IL-1β production (25). These previous reports are summarized in Table 1 and Figure 1. However, kinases related to NLRP3 tyrosine phosphorylation remain to be identified.

**Table 1:** Regulation of NLR family pyrin domain (PYD)-containing 3 (NLRP3) inflammasome activation through posttranslational modification (PTM) of NLRP3.

PTM	Modification site	Enzymes/triggers	Effect on NLRP3 inflammasome activation	Reference
Phosphorylation	Y861 (human) Y859 (mouse)	Protein tyrosine phosphatase non-receptor 22	Dephosphorylation of NLRP3 (⇧)	(25)
	S295 (human) S291 (mouse)	Protein kinase A	Phosphorylation of NLRP3 (⇩)	(26, 27)
	S5 (human) S3 (mouse)	Protein phosphatase 2A	Dephosphorylation of NLRP3 (⇧)	(28)
	S198 (human) S194 (mouse)	Jun N-terminal kinase	Phosphorylation of NLRP3 (⇧)	(29)
	S295 (human) S293 (mouse)	Protein kinase D	Phosphorylation of NLRP3 (⇧)	(30)

Ubiquitination	Leucine-rich repeat (LRR) domain	BRCC3 (mouse) BRCC36 (human)	Deubiquitination of NLRP3 (⇧)	(40)
	K689 (human)	F-box L2	Ubiquitination of NLRP3 (↓, ⇩)	(42)
	Pyrin domain	TRIM31	Ubiquitination of NLRP3 (↓, ⇩)	(43)
	Nucleotide-binding domain (NBD) and LRR domain	MARCH7	Ubiquitination of NLRP3 (↓, ⇩)	(44)
	NBD	Ariadne homolog 2	Ubiquitination of NLRP3 (⇩)	(41)

Alkylation	C419 (human)	Acrylamide derivatives	Alkylation of NLRP3 (⇩)	(51)

S-nitrosylation	Unknown	Nitric oxide and SNAP	S-nitrosylation of NLRP3 (⇩)	(54)

*↓, Decrease of NLRP3 protein level; ⇧, activation of NLRP3 inflammasome; ⇩, inhibition of NLRP3 inflammasome*.

### Protein Kinase A (PKA)

Protein kinase A is a serine/threonine kinase important in controlling the immune system by modulating gene transcription and post-translational signaling (35). Recently, Sokolowska et al. suggested that PKA inhibits NLRP3 inflammasome activation through the induction of NLRP3 serine phosphorylation (36). Prostaglandin E2 (PGE2) stimulates the E-prostanoid 4 (EP4) receptor and activates PKA signaling by increasing cytoplasmic cyclic adenosine monophosphate (cAMP) levels (36). PGE2-mediated EP4 signaling causes PKA–NLRP3 interactions, and subsequently activates PKA, then phosphorylates NLRP3 within the NBD at S295 by increasing cytoplasmic cAMP levels (Table 1; Figure 1). Phosphorylation of the S295 residue in NLRP3 negatively regulates NLRP3 inflammasome activation through reduction of the ATPase activity of NLRP3 (26). Bile acids also phosphorylate the S295 residue in NLRP3 through the cAMP–PKA axis (27). Bile acids stimulate transmembrane G protein-coupled receptor-5 (TGR5), TGR5 signaling activates PKA through increasing cytoplasmic cAMP levels, and activated PKA further induces phosphorylation of the S295 residue in NLRP3, leading to NLRP3 inhibition. Bile acid and TGR5 signaling promotes mixed K63 and K48 ubiquitination of NLRP3 through phosphorylation of the S295 residue in NLRP3 (27). Forskolin, a PKA activator that increases cytoplasmic cAMP levels inhibits NLRP3 inflammasome activation by phosphorylation of the S295 residue in NLRP3. Several CAPS mutations in the region between residues I290 and D311 show that phosphorylation of the S295 residue in NLRP3 are impaired in CAPS-mutant NLRP3 (26). In an *in vivo* experiment, PKA activation attenuated both LPS-induced sepsis and alum-induced peritonitis *via* suppression of NLRP3 inflammasome activation. Further, PKA activation improved insulin sensitivity in high-fat diet-induced type-2 diabetes through suppression of NLRP3 inflammasome activation (27).

### Protein Phosphatase 2A (PP2A)

Phosphorylation of the S5 residue in the PYD of NLRP3 negatively regulates NLRP3 inflammasome activation. Phosphorylation of the S5 residue in NLRP3 causes electrostatic repulsion of PYDs. PP2A promotes NLRP3 inflammasome activation through dephosphorylation of the S5 residue in NLRP3 (Table 1; Figure 1). Okadaic acid, a PP2A inhibitor, significantly attenuates NLRP3 inflammasome activation. NLRP3 inflammasome activation is impaired in PP2A-deficient macrophages (28). However, Martin et al. suggested that PP2A is also related to localization of ASC through the regulation of ASC phosphorylation *via* decreasing IKKα kinase activity in a PP2A-dependent manner (37). According to their study, PP2A induced dephosphorylation of IKKα through the interaction with IKKα in NLRP3 inflammasome-activated macrophages (37). For this reason, the specificity of PP2A is not yet clear, and the kinase involved in the phosphorylation of the S5 residue in NLRP3 remains to be identified.

### Jun N-Terminal Kinase (JNK)

C-Jun N-terminal kinase-mediated NLRP3 phosphorylation is an essential priming event for NLRP3 inflammasome activation. NLRP3 is directly phosphorylated by JNK1 during priming, but not by JNK2. All toll-like receptor ligands phosphorylate the S194 residue in NLRP3 in a JNK1-dependent manner (Table 1; Figure 1). JNK1-mediated NLRP3 phosphorylation promotes the deubiquitination of NLRP3 and facilitates the self-association of NLRP3. NLRP3 inflammasome activation is completely inhibited in JNK1-deficient macrophages, and JNK inhibitors also completely inhibit NLRP3 inflammasome activation, but do not inhibit AIM2 or NLRC4 inflammasome activation. NLRP3 S194A mutants fail to activate the NLRP3 inflammasome both *in vitro* and *in vivo*. S194A-mutant NLRP3 expressed in mice prevented both MSU-induced peritonitis and LPS-induced sepsis through inhibition of NLRP3 inflammasome activation (29).

### Protein Kinase D

Protein kinase D (PKD)-mediated NLRP3 phosphorylation is required for full activation of the NLRP3 inflammasome. During the NLRP3 inflammasome activation process, NLRP3 directly binds to mitochondria-associated membranes (MAMs) and NLRP3 protein level increases in MAMs fraction (38, 39). Zhang et al. suggested that during NLRP3 inflammasome activation process, MAMs localized to adjacent Golgi membrane and diacylglycerol (DAG) increased at the Golgi (30). As DAG accumulation at Golgi occurred at upstream of NLRP3 inflammasome activation, deletion of NLRP3 could not affect the DAG accumulation at Golgi. DAG accumulation at Golgi triggers the activation of PKD, a key effector of DAG activation. Activated PKD subsequently interacts with NBD of NLRP3 and phosphorylates the S293 residue in NLRP3. PKD-mediated NLRP3 phosphorylation leads to release of NLRP3 from MAMs to cytoplasm, allowing full NLRP3 inflammasome maturation. Both inhibition of PKD activity and PKD-deficient macrophages resulted in decrease of NLRP3 inflammasome activation which is due to the inhibition of NLRP3 release from MAMs to cytoplasm. PKD inhibition prevents NLRP3 inflammasome activation in peripheral blood mononuclear cells from CAPS patients. PKD inhibitor also prevents both LPS-induced systemic inflammation and *S. aureus*-induced inflammation in *in vivo* through the inhibition of NLRP3 inflammasome activation (30).

## NLRP3: Ubiquitination

NLR family pyrin domain (PYD)-containing 3 ubiquitination was first reported by Py et al. (40), and two different kinds of NLRP3 ubiquitination-mediated negative regulation of NLRP3 have been reported: mixed K63 and K48 ubiquitination-mediated NLRP3 inactivation (40, 41) and K48 ubiquitination-mediated proteasomal degradation of NLRP3 (42–44). The drugs that increase ubiquitinated-NLRP3 population, such as G5 were widely used to inhibit NLRP3 inflammasome activation (40).

### BRCC3

Deubiquitination of NLRP3 is essential to NLRP3 inflammasome activation, and some deubiquitinase inhibitors negatively regulate NLRP3 inflammasome activation. The priming signal of LPS stimulation is important not only for increasing expression levels of NLRP3 inflammasome components, such as NLRP3 and pro-IL-1β, but also for inducing NLRP3 deubiquitination, which puts NLRP3 in its ready state (40, 45). Py et al. suggested that the deubiquitinase BRCC3 played an important role in NLRP3 inflammasome activation through deubiquitination of NLRP3 and interaction with the LRR domain of NLRP3 (Table 1; Figure 1). Inhibition of NLRP3 deubiquitination by G5, a BRCC3 inhibitor, increased the polyubiquitination of NLRP3 with mixed K63 and K48 chains, which critically inhibited NLRP3 inflammasome activation, and this ubiquitination was not related to protein degradation (40). During the NLRP3 inflammasome activation process, both the priming and activation signal can induce deubiquitination of NLRP3, and these two independent deubiquitination signals are important to NLRP3 inflammasome activation. Priming signal-mediated NLRP3 deubiquitination is induced in a reactive oxygen species-dependent manner, but the mechanism activating signal-mediated NLRP3 deubiquitination is unknown (46). A recent study suggested that kinase activity of JNK, which is a representative stress signal, was essential to priming signal-mediated NLRP3 deubiquitination through induction of NLRP3 phosphorylation (29). However, the exact ubiquitination site, ubiquitin E3 ligase, and mechanism activating signal-mediated deubiquitination of NLRP3 remain to be identified.

### F-Box L2 (FBXL2)

Protein degradation-induced ubiquitination has also been detected in NLRP3. A recent study suggested that the Skp-Cullin-F-box (SCF) family member, FBXL2, which is a ubiquitin E3 ligase, directly interacted with NLRP3, leading to proteasomal degradation of NLRP3 through ubiquitination. FBXL2 recognizes and interacts with the tryptophan-73 residue within NLRP3, and then subsequently ubiquitinates the lysine-689 residue, leading to NLRP3 degradation (Table 1; Figure 1). FBXL2 regulates stability of the NLRP3 protein through ubiquitination-mediated proteasomal degradation. The LPS priming signal increases stability of the NLRP3 protein through reducing FBXL2 levels. LPS activates F-box O3 (FBXO3), and the activated FBXO3 ubiquitinates and degrades FBXL2. BC-1215, an FBXO3 inhibitor, significantly inhibits NLRP3 inflammasome activation by decreasing NLRP3 expression levels *via* increasing FBXL2 expression levels (42). The LPS priming signal is important not only to increase NLRP3 protein levels but also to extend the half-life of the NLRP3 protein, creating a suitable environment for NLRP3 inflammasome activation. However, whether BRCC3 can deubiquitinate the K689 residue in NLRP3 through FBXL2 is unclear.

### TRIM31

TRIM31, an E3 ubiquitin ligase, also causes K48-linked ubiquitination of NLRP3, leading to its proteasomal degradation and thus reducing inflammasome activation. TRIM31 directly interacts with the PYD of NLRP3 (Table 1; Figure 1). Intracellular TRIM31 expression levels increase by stimulation with either LPS or IL-1β in macrophages, suggesting that TRIM31 is a part of feedback suppressor of the NLRP3 inflammasome through reduction in NLRP3 expression. In an *in vivo* experiment, TRIM31-deficient mice exhibited higher concentrations of IL-1β in the serum, but not of TNF-α or IL-6 in an LPS-induced peritonitis model. The mice also showed increased recruitment of neutrophils and monocytes to the peritoneum in alum-induced peritonitis through hyperactivation of the NLRP3 inflammasome. These data suggested that TRIM31-mediated ubiquitination and degradation of NLRP3 negatively regulated NLRP3 inflammasome activation (43).

### MARCH7

Dopamine, which functions as a neurotransmitter in the brain, inhibits NLRP3 inflammasome activation through MARCH7-mediated ubiquitination and autophagic degradation of NLRP3 in response to stimulation of the dopamine D1 receptor (DRD1) (44). Although the dopamine concentration (approximately 100 µM) required for NLRP3 inflammasome inhibition *in vitro* is higher than the reported physiological concentration of dopamine (approximately 1 µM) (47, 48), dopamine-mediated DRD1 signaling induced degradation of NLRP3, but not other inflammasome components, and this degradation was prevented by autophagic inhibition, but not by inhibition of proteasomal degradation. DRD1 signaling increases cytoplasmic cAMP levels, which in turn cause not only MARCH7-dependent K48-linked ubiquitination but also autophagic degradation of NLRP3. MARCH7 reportedly binds both the NBD and LRR domains of NLRP3 (Table 1; Figure 1). Dopamine does not inhibit NLRP3 inflammasome activation in MARCH7-deficient macrophages. Dopamine-mediated DRD1 signaling prevents MPTP-induced neurodegeneration through suppression of the NLRP3 inflammasome. Dopamine also prevents MPTP-induced dopaminergic neuronal cell death and reduced NLRP3 expression levels. However, dopamine does not prevent MPTP-induced neurodegenerative disease in DRD1-deficient mice (44). Dopamine-mediated DRD1 signaling also prevents both LPS-induced systemic inflammation and MSU-induced peritonitis through suppression of IL-1β production *via* inhibition of NLRP3 inflammasome activation, but not by suppression of TNF-α (44).

### Ariadne Homolog 2 (ARIH2)

Ariadne homolog 2, an E3 ubiquitin ligase, negatively regulates NLRP3 inflammasome activation by inducing mixed K63 and K48 ubiquitination of NLRP3. The RING2 domain of ARIH2 is critical for the interaction with the NBD of NLRP3 (Table 1; Figure 1). Further, ARIH2 is co-localized with NLRP3 inflammasome components, such as NLRP3 and ASC, in NLRP3 inflammasome-activated macrophages. ARIH2-deficient macrophages exhibit increased NLRP3 inflammasome activation through reduction of NLRP3 ubiquitination. On the contrary, ARIH2-overexpressing macrophages prevent NLRP3 inflammasome activation by increasing NLRP3 ubiquitination. Thus, ARIH2 is an endogenous posttranslational negative regulator of NLRP3 inflammasome activation in macrophages (41).

## NLRP3: Alkylation

NLR family pyrin domain (PYD)-containing 3 alkylation was first reported by Juliana et al. (49), and some NLRP3 inflammasome-specific inhibitors, including Bay11-7082, 3,4-methylenedioxy-β-nitrostyrene (MNS), some acrylamide derivatives, and 2-cyclohexylimino-6-methyl-6,7-dihydro-5H-benzo[1,3]oxathiol-4-one (BOT-4-one) work *via* direct alkylation of NLRP3. These chemicals commonly have an electrophilic region, and this region forms covalent bonds with NLRP3 (49–51). Cocco et al. predicted the Cys419 residue in the ATPase catalytic pocket of NLRP3 as the plausible alkylation site for acrylamide derivatives *via* computational analysis (Table 1; Figure 1) (51). The functional role of NLRP3-alkylating agents commonly appears to be critically mediated by inhibition of NLRP3 ATPase activity. NLRP3 alkylation reduces the ATP-binding affinity of NLRP3 (49), thereby resulting in impaired ATPase activity (50, 51). The ATPase activity of NLRP3 is important both to NLRP3 self-association and to NLRP3–ASC association (52). Recently, Shim et al. suggested that NLRP3 alkylation is also related to ubiquitination of NLRP3. NLRP3-alkylating agents, such as Bay11-7082, MNS, and BOT-4-one, increase ubiquitinated NLRP3 levels through their alkylating activity (53). Some electrophilic chemicals that mediate NLRP3 alkylation negatively regulate NLRP3 inflammasome activation, but the exact alkylation site in NLRP3 should be identified by crystallization and mass spectrometry.

## NLRP3: S-Nitrosylation

Nitric oxide (NO) negatively regulates NLRP3 inflammasome activation through S-nitrosylation of NLRP3. NO is important to the inhibition of bacterial growth and can be induced by T cell-derived interferon-γ (IFN-γ) in macrophages. Both IFN-γ-induced NO production and NO donor (SNAP) treatment inhibit canonical and *Mycobacterium tuberculosis*-mediated NLRP3 inflammasome activation through induction of NO-induced S-nitrosylation of NLRP3 (Table 1; Figure 1) (54). However, the exact S-nitrosylation site of NLRP3 remains to be identified.

## Perspective

Here we focused on the role of PTMs of NLRP3 in NLRP3 inflammasome activation. NLRP3 is clearly subjected to various PTMs involved in the precise activation of the NLRP3 inflammasome. PTMs of NLRP3 may be good targets for the development of NLRP3-specific drugs or inhibitors. However, the exact PTM sites of NLRP3 and PTM-related enzymes remain to be identified, and the correlations and interactions among various types of PTMs of NLRP3 warrant future investigation.

## Author Contributions

D-WS drafted the manuscript, figure, and table. K-HL supervised and edited the manuscript, figure, and table.

## Conflict of Interest Statement

The authors declare that the research was conducted in the absence of any commercial or financial relationships that could be construed as a potential conflict of interest.
